# Skin-Friction-Based Identification of the Critical Lines in a Transonic, High Reynolds Number Flow via Temperature-Sensitive Paint

**DOI:** 10.3390/s21155106

**Published:** 2021-07-28

**Authors:** Marco Costantini, Ulrich Henne, Christian Klein, Massimo Miozzi

**Affiliations:** 1 German Aerospace Center (DLR), Institute of Aerodynamics and Flow Technology, Bunsenstrasse 10, D-37073 Göttingen, Germany; Ulrich.Henne@dlr.de (U.H.); Christian.Klein@dlr.de (C.K.); 2 National Research Council (CNR), Institute of Marine Engineering, Via di Vallerano 139, I-00128 Rome, Italy; Massimo.Miozzi@cnr.it

**Keywords:** skin friction, temperature-sensitive paint, separation, reattachment, transonic flow, shock-wave/boundary-layer interaction

## Abstract

In this contribution, three methodologies based on temperature-sensitive paint (TSP) data were further developed and applied for the optical determination of the critical locations of flow separation and reattachment in compressible, high Reynolds number flows. The methodologies rely on skin-friction extraction approaches developed for low-speed flows, which were adapted in this work to study flow separation and reattachment in the presence of shock-wave/boundary-layer interaction. In a first approach, skin-friction topological maps were obtained from time-averaged surface temperature distributions, thus enabling the identification of the critical lines as converging and diverging skin-friction lines. In the other two approaches, the critical lines were identified from the maps of the propagation celerity of temperature perturbations, which were determined from time-resolved TSP data. The experiments were conducted at a freestream Mach number of 0.72 and a chord Reynolds number of 9.7 million in the Transonic Wind Tunnel Göttingen on a VA-2 supercritical airfoil model, which was equipped with two exchangeable TSP modules specifically designed for transonic, high Reynolds number tests. The separation and reattachment lines identified via the three different TSP-based approaches were shown to be in mutual agreement, and were also found to be in agreement with reference experimental and numerical data.

## 1. Introduction

Improvements in the aerodynamics of commercial aircraft, and in particular in the reduction of drag [1,2], are needed in order to reach the targets in polluting emission reduction set by the European Commission [3] and by NASA [4]. Since skin-friction drag is the major source of drag (contributing about half of the total aircraft drag [4,5]), substantial friction drag reduction can be achieved by maintaining the flow laminar over large portions of the aircraft surfaces [5,6]. However, laminar boundary layers are also more prone to separation than their turbulent counterparts [7,8]. Flow separation may have a negative impact on the aerodynamic performance of the aircraft surfaces, especially when induced by a Shock-Wave/Boundary-Layer Interaction (SWBLI) [7,8,9,10,11]. This complex phenomenon [11,12,13] is likely to occur at transonic flow conditions on aircraft wings designed with laminar flow technology, where the laminar boundary layer over the suction side of the wing reaches supersonic speeds; the supersonic flow region is typically terminated by a (normal) shock, which interacts with the boundary layer and induces flow separation [1,14,15]. In this case, the flow generally undergoes transition to turbulence, and the strongly increased wall-normal transport of momentum (and energy) eventually leads to the reattachment of the turbulent flow to the surface. The resulting region of reverse flow enclosed within the boundary layer is commonly referred to as a Laminar Separation Bubble (LSB) [7,8,10,14,16,17]. Laminar SWBLI can also occur in other aerodynamic applications, including internal flows in the gas turbine engines of transonic commercial aircraft [7,18,19] and in the engines of supersonic and hypersonic vehicles [8,10,16]. Major concerns arise in the presence of laminar SWBLI because it can cause detrimental effects for the aircraft performance, such as drag increase, flow unsteadiness, local heat peaks, high aerodynamic loads and increased structural fatigue of the aircraft components [7,8,9,10,16,19]. From these considerations, it appears clear that accurate information on the occurrence of a laminar separation bubble induced by SWBLI, including the locations of flow separation and reattachment over the whole examined surface, is crucial in a variety of aerodynamic applications. This information is essential not only for the direct quantification of the performance of the aerodynamic technologies, but also for the validation of numerical tools, especially those providing the foundations for the comprehensive digital description and development of flying vehicles [20].

Among all of the quantities relevant in compressible flows, skin friction appears to be the most appropriate for the global identification of boundary-layer separation and reattachment, since skin-friction fields allow for a physics-based identification of the loci of flow separation and reattachment, i.e., of the critical points and lines (see [21,22,23,24,25,26], among others). In an essentially two-dimensional flow, the critical lines can be identified at the locations where the streamwise component of the skin-friction vector is zero; starting on mass conservation, the separation and reattachment lines are more generally identified at the locations of, respectively, convergence and divergence of the skin-friction lines.

On the other hand, skin friction is also regarded as “the most difficult surface quantity to measure” [21]. A thorough review of the variety of techniques developed and applied for the measurement of the skin friction is beyond the scope of this introduction, which reports only an overview oriented towards their application in compressible, high Reynolds number flows. Reviews of the measurement techniques developed in the past can be found in, e.g., [27,28,29,30]. Floating elements [31] provide an average measurement of the skin friction over a certain area, but clearly not the distribution of the skin-friction field. Localized skin-friction data can be obtained via micro-floating element sensors based on Micro-Electro-Mechanical Systems (MEMS) [27,32], via hot films [33,34] and via wall hot wires [35], but these single, surface-based sensing elements have to be arranged in arrays to obtain a skin-friction distribution, which still has limited spatial resolution. Moreover, the required complexity of the arrays increases for the determination of the direction of the skin-friction vector, and the application of all these surface-mounted sensors in high Reynolds number flows is particularly difficult because of their relatively large size compared to the small thickness of the boundary layer. This is also one of the limitations [30] for the use of Preston tubes [36]. The skin-friction magnitude and direction can be obtained from the measurements of the velocity profiles within the boundary layer. These can be accomplished by means of traversable hot wires [30] or via velocimetric techniques, such as Laser Doppler Velocimetry (LDV) [28,30], Particle Image Velocimetry (PIV) [37] and Lagrangian Particle Tracking (LPT) [38]. Besides the issues related to a traversing mechanism for hot wires, these measurement techniques can generally provide accurate velocity profiles in a wall-bounded flow only for a limited volume. Moreover, the measurement of the velocity gradients close to the wall is very challenging, so that the measurable velocity profiles are often processed via correlation methods (such as the Clauser plot method [39]) to determine the skin friction.

Global skin-friction measurements can be performed via surface-based optical methods. Techniques based on the measurement of the development of an oil film applied to the surface of interest, such as Oil-Film Interferometry (OFI) [28,40,41,42,43], Particle Image Surface Flow Visualization (PISFV) [44,45,46] and the Global Luminescent Oil Film (GLOF) [21,47,48] methods, allow for the quantitative determination of the skin-friction field. As a more qualitative technique, surface oil-flow visualization can also be a useful tool for the estimation of the locations of critical points and lines (see, e.g., [19,49]). A limiting drawback of all oil-film-based techniques is the need for oil re-application after each wind-tunnel run. Moreover, the oil-film based techniques cannot be applied at cryogenic conditions, and their application is obviously challenging in flight tests [50]. The Micro-Pillar Shear-Stress Sensor (MPS^3^) [51,52] also enables global skin-friction measurements via a high-resolution array of micro-pillars, flush-mounted on the surface of interest. Besides the necessary surface preparation, the requirements on the micro-pillar geometry and materials currently limit their application in air flows to moderate Reynolds numbers. In principle, pressure and skin-friction fields can be obtained simultaneously from the normal and tangential components of a Surface Stress Sensitive Film (S^3^F) [53], but this technique has not been applied in compressible, high Reynolds number flows yet. A major challenge for non-intrusive testing at these flow conditions may be the integration of the S^3^F into the surface of the wind-tunnel models. Skin-friction fields can be also determined via Shear-Sensitive Liquid Crystals (SSLC) [9,54,55], but the strong sensitivity of SSLC to lighting and viewing directions, combined with complicated data analysis, generally limits their application in aerodynamic studies [21,28].

The above discussion is far from being exhaustive, but it provides an indication of the critical need of further developments in the experimental methods for the global measurement of the skin friction in compressible, high Reynolds number flows, especially for the detection of flow separation and reattachment. The recent developments in the analysis of thermographic data, in particular those measured via the Temperature-Sensitive Paint (TSP) technique [24,25,26,56,57,58], show the potential for the experimental determination of the skin-friction fields even at challenging flow conditions. A first approach is grounded on the relation between the skin-friction vector and the surface temperature gradient contained in the energy equation at the wall [56]. A second methodology relies on the link between the propagation celerity of temperature perturbations and the friction velocity [25,57,58]. These TSP-based methodologies have been successfully applied to identify the critical lines in low-speed flows [24,25,26,56,57,58,59,60].

In this work, the TSP-based methodologies were adapted and applied for the first time in a compressible, high Reynolds number flow. The investigations were performed in the Transonic Wind Tunnel Göttingen on the VA-2 supercritical airfoil model [48,61,62], focusing on the model upper surface. The examined test conditions were a freestream Mach number M = 0.72, a chord Reynolds number Re = 9.7 × 10^6^, and an angle of attack AoA = 1.5°, which enabled the achievement of transonic flow conditions on the model upper surface and thus the study of flow separation and reattachment resulting from a laminar SWBLI. The modular construction of the investigated wind-tunnel model allowed for the installation on the model upper side of two exchangeable inserts, which were specifically designed for the application of TSP at the considered flow conditions. The model was also equipped with pressure taps and thermocouples in order to obtain supporting information for the TSP-based analysis. The TSP data were acquired at both low and high frequencies, and were analyzed via the skin-friction extraction methodologies introduced above. The critical lines determined by means of the TSP-based approaches were compared with reference data from experimental [48] and numerical investigations. Finally, the feasibility of obtaining quantitative skin-friction distributions from the TSP data at the examined transonic flow conditions was critically explored.

## 2. Applied Methods

### 2.1. Temperature-Sensitive Paint Measurement Technique

The working principle of TSP relies on the thermal quenching process of temperature-sensitive molecules (luminophores) embedded in a binder material, which is applied as a coating on the surface of interest. The luminophores can be excited by the absorption of light in an appropriate (luminophore-specific) wavelength range, and one of the mechanisms for their return to the electronic ground state is the emission of light, which occurs at a higher wavelength than that of the excitation light (Stokes shift). The intensity of the emitted light decreases at higher temperatures, and this property is used to measure the surface temperature distribution via TSP [63,64,65].

In several applications, including underwater investigations and wind-tunnel experiments at subsonic to low supersonic Mach numbers, the natural surface temperature variations (mainly induced by the action of the skin friction) are typically too small to be detected via TSP. For this reason, an artificial enhancement of the temperature differences at the surface is needed; this is accomplished by imposing a heat flux at the model surface [63,64]. Since the forced-convection heat transfer coefficient is generally a function of the skin friction, the imposed flow-surface temperature difference is transferred at different rates depending on the local skin friction, thus leading to augmented surface temperature variations that can be measured by means of TSP. The surface heat flux can be imposed through a variety of methods reported in previous work [25,60,63,64,65,66,67,68,69,70,71,72,73].

In this work, the surface heat flux was imposed via two different types of electrical heating systems integrated beneath the TSP: a layer of Carbon NanoTubes (CNT) [65,70,71,72] and a current-carrying carbon fiber layer [64,73,74]. The integration of these electrical heating layers in the TSP layer composition was specifically designed for the examined test conditions in a transonic wind tunnel, as described in Section 3.2.

### 2.2. Skin-Friction Extraction Methodologies

Two different methodologies have been presented in recent years to generally determine skin-friction fields from surface temperature distributions measured via TSP, even in the presence of separated flows.

The first approach relies on the relationship between skin friction and wall heat flux described by the energy equation at the wall. The resulting equation can be seen as a differential, generalized form of the Reynolds analogy, which is a well-known analogy to empirically describe the relationship between skin friction and wall heat flux. In most flow scenarios, however, this relationship is more complex than the description provided by the Reynolds analogy (or by similar analogies), which cannot be applied in such cases– especially when flow separation occurs. In contrast, the general approach presented in this work allows for the extraction of skin-friction fields even in complex flow scenarios (separated flows included). It is discussed in Section 2.2.1, focusing on its application for transonic, high Reynolds number flows.

The second methodology is based on the observations of the near-wall behavior of the perturbations of flow quantities, and in particular of the temperature, which is assumed to behave as a passive scalar. Far from a rigid surface, a passive scalar is transported by a moving fluid at the same (mean) velocity as the surrounding flow. In the vicinity of a rigid surface, however, the mean velocity approaches zero, while perturbations of the flow quantities propagate at a celerity proportional to the friction velocity. Since this behavior is similar to that of a wave, the term “celerity” is used in this work to define the propagation velocity of the perturbations of flow quantities, as is consistent with [57,58,75]. Two approaches grounded on the determination of the propagation celerity of temperature perturbations from time-resolved TSP data, and on the relationship between this propagation celerity and the friction velocity, are discussed in Section 2.2.2.

#### 2.2.1. Approach Based on the Energy Equation at the Wall (OF Approach)

The first method to experimentally determine skin-friction fields from surface temperature measurements was presented in [56,59]. In this method, the relationship between a skin-friction field and a surface temperature field is derived from the energy equation at the wall, which is recast in a form similar to that of the Optical Flow (OF) equation [76], as described below.

The expansion in Taylor series of the velocity and temperature terms yields the following form of the energy equation at the wall:

(1) $$F+\tau_{w,x}\partial T_{w}/\partial x+\;\tau_{w,y}\partial T_{w}/\partial y\;=0$$

where *x* and *y* are the coordinates in the streamwise and spanwise directions, respectively, *t* is the time, ${\vec{\tau }}_{w}$(*x*,*y*,*t*) is the wall shear stress, *T_w_*(*x*,*y*,*t*) is the surface temperature, and *F* is a source term, which contains the contributions of heat-flux time rate, thermal diffusion and viscous dissipation. Equation (1) provides a relationship between skin-friction field and temperature gradients at the wall, modulated by a forcing term. It represents a balance between the skin-friction vector, projected on the normal vector ∇*T_w_* to an iso-temperature line (*T_w_* = const.), and the source term *F*. When the surface temperature field is available (for example, via TSP measurements), the skin-fiction vector ${\vec{\tau }}_{w}$ can be extracted by introducing a regularization term (with the lagrangian multiplier *α*) in Equation (1) and solving the corresponding ill-posed problem with an inverse procedure based on a variational approach, thus obtaining the relative (normalized) ${\vec{\tau }}_{w}$-field. This approach relies on image-based temperature measurements, in which Equation (1) is projected onto the image plane of a camera. Since the recast form of Equation (1) is similar to the OF equation [76] (with the optical flow and the time derivative of the image intensity replaced by the projected ${\vec{\tau }}_{w}$-vector and by the source term *F*, respectively), this method will be named the “OF approach” in this work. Note here that the spatial temperature gradients in the regularization term, required by the variational approach [56,59], introduce an expansion of the space metric rank, thus allowing for the step from a scalar function of the temperature to a vector function of the skin friction.

Since the source term in Equation (1) is very difficult to measure, a heuristic model for *F* was proposed in [56], as:

(2) $$F=\eta \left(T_{w}-T_{ref}\right)+\varepsilon$$

where *η* is an empirical constant (linked to the heat transfer coefficient), *T_ref_* is an appropriate reference temperature, and ε is a term modeling all of the remaining contributions to *F* and determined iteratively. The absolute ${\vec{\tau }}_{w}$-field can be determined from the relative skin-friction field obtained via the OF approach when reference data (at least localized) are available, thus allowing for a calibration of the used parameters [56,59].

The OF approach has been successfully applied to obtain skin-friction fields in incompressible flows [24,25,26,56,58,59,60,75]. At these flow conditions, the temperature difference (*T_w_*−*T_ref_*) had to be augmented via the heat-flux enhancement methodologies described in Section 2.1. In practice, the measured surface temperature distribution *T_w_* was the temperature of the heated surface, whereas the reference temperature *T_ref_* was reasonably assumed to be uniform and equal to the ambient / outer flow temperature with an inactive heating system. This assumption was valid especially in underwater applications [24,25,57,58,60,75]. In compressible flows, however, this assumption is no longer possible, because the effect of the dissipation within the boundary layer already induces a surface temperature distribution on an adiabatic wall [77]. Nevertheless, if this adiabatic-wall temperature distribution *T_aw_* can be measured, it provides the appropriate reference temperature *T_ref_* for Equation (2) in compressible flows. This approach was pursued in the present work. Both temperature maps, at adiabatic-wall (*T_aw_*(*x*,*y*) = *T_ref_*) and heated-wall conditions (*T_w_*(*x*,*y*)), were measured via TSP, according to the procedure described in Section 4. In this manner, the OF approach was applied for the first time in a compressible, high Reynolds number flow, enabling the determination of the topology of the skin-friction lines on the investigated surface, and thus also of the flow separation and reattachment locations. As introduced in Section 1 and discussed in [24,25,26,59,60,75], the skin-friction lines determined by means of the OF approach provide a physics-based criterion for the identification of the critical lines.

#### 2.2.2. Approaches Based on the Celerity of Propagation of Temperature Perturbations

A novel methodology for the measurement of the skin friction from time-resolved TSP data was presented in [25,57,75]. It is based on the observations reported in the literature about the direct relationships existing between the friction velocity ${\vec{u}}_{\tau }$ and the propagation celerity of velocity perturbations (see [78,79], among others), as well as between the propagation celerities of velocity and temperature perturbations [80].

According to the relationships from [78,79,80], the friction velocity can be evaluated when the propagation celerity of temperature perturbations ${\vec{U}}_{T}$ is known. As discussed in [25,57,58,75], the traces of the temperature fluctuations at the wall ($T_{w}^{\prime }$) can be measured via TSP when enhanced by one of the methodologies to impose a heat flux at the investigated surface (see Section 2.1). The analysis of the time-resolved $T_{w}^{\prime }$-maps obtained from the TSP data can provide maps of ${\vec{U}}_{T}$, thus enabling the determination of skin-friction fields through the relationship between ${\vec{U}}_{T}$ and ${\vec{u}}_{\tau }$. In essentially two-dimensional flows, separation and reattachment lines can be directly identified at the locations where the propagation celerity of temperature perturbations becomes zero. Two approaches to determine ${\vec{U}}_{T}$ from time-resolved TSP data were presented in [57,58,75], and are summarized below.

- Minimization of the dissimilarity from the Taylor Hypothesis (TH approach).

The validity for flowfield regions close to the wall of the assumption known as the Taylor hypothesis of “frozen turbulence” [81] was investigated in [82,83], among others. The main findings restricted the applicability of the Taylor hypothesis to locations far from the wall, while the propagation celerity of velocity perturbations close to the wall becomes nearly a constant, proportional to the local friction velocity (see above). The approach proposed in [82,83] for the propagation celerity of velocity and vorticity perturbations was adapted to surface temperature fluctuations in [57,58,75]. Following this approach, ${\vec{U}}_{T}$ can be determined by assuming that the surface temperature fluctuations behave as traceable passive scalars, which propagate at the celerity ${\vec{U}}_{T}$ that minimizes the dissimilarity of the observed behavior with that conform to the Taylor hypothesis. In practice, for an essentially two-dimensional flow, where ${\vec{U}}_{T}$ = $U_{T}$ is the streamwise component of the celerity of propagation of temperature fluctuations, this condition corresponds to [57,58,75]:

(3) $$min\left\{\bar{{\left|-\frac{\partial T_{w}^{\prime }}{\partial t}-U_{T}\frac{\partial T_{w}^{\prime }}{\partial x}\right|}^{2}}\right\}$$

Equation (3) is a modified version of the mean square error proposed in [82], which minimum condition provides:

(4) $${\tilde{U}}_{T}=\frac{\bar{\left|\left(-\frac{\partial T_{w}^{\prime }}{\partial t}\right)\;\frac{\partial T_{w}^{\prime }}{\partial x}\right|}}{\bar{{\left|\frac{\partial T_{w}^{\prime }}{\partial x}\right|}^{2}}}$$

where ${\tilde{U}}_{T}\equiv \left|U_{T}\right|$. Separation and reattachment positions correspond to the locations of the zero-crossing of $U_{T}$; with the consideration of the modulus in ${\tilde{U}}_{T}$, they are identified at the locations of the (sharp) minima of the correlation function (Equation (3)), where:

(5) $$\frac{\partial }{\partial x}\left({\tilde{U}}_{T}\bar{\frac{\partial T_{w}^{\prime }}{\partial x}}\right)=0$$

In this work, the TH approach was applied for the first time to a compressible flow.

- Tracking of thermal perturbations (TR approach).

A second approach for the determination of the propagation celerity of temperature perturbations ${\vec{U}}_{T}$ was recently presented in [58]. It is based on the direct tracking of the wall temperature perturbations via an efficient optical flow algorithm, which relies on the Dense Inverse Search algorithm (DIS) proposed in [84]. The three main steps performed by the algorithm are:

o Fast inverse search for sparse correspondences according to an optimized inverse procedure [85] based on the Lucas-Kanade technique [86];
o Densification to compute a dense flow field;
o Variational refinement of the dense flow field.

The DIS optical flow algorithm allows the extraction of robustly estimated dense flows between couples of images at a very high computational speed, and thus obtaining the propagation celerity of temperature perturbations with limited computational time even for a very large TSP dataset. This novel approach was applied here for the first time to a compressible flow.

Concluding this section, it should be emphasized that the TH approach is fed by a set of $T_{w}^{\prime }(x,y$) maps and returns the time-averaged distribution of $\left|U_{T}\left(x,y\right)\right|$, while the TR approach requires pairs of $T_{w}^{\prime }(x,y$) maps separated by a certain time difference Δ*t* and returns a time series of $U_{T}\left(x,y\right)$.

## 3. Experimental Setup

### 3.1. Transonic Wind Tunnel Göttingen

**Figure 1:**
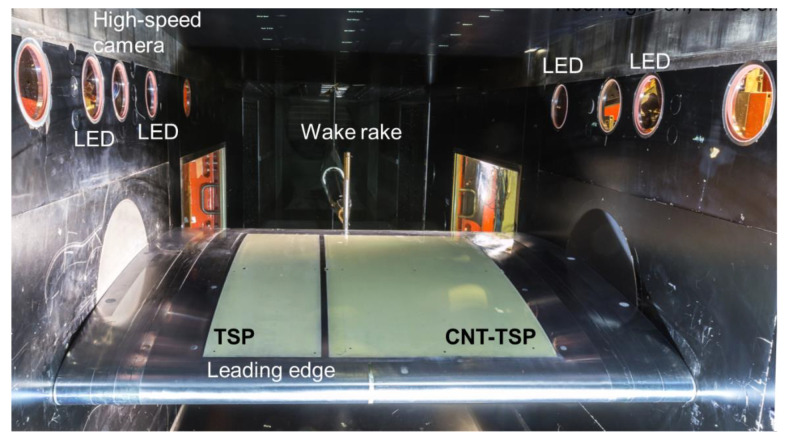
VA-2 supercritical airfoil model (with CNT-insert, see Section 3.2.1) and measurement techniques installed in the DNW-TWG test section. Light-Emitting Diodes (LEDs) and cameras are behind the indicated windows. Room light on, LEDs off.

The experiments were conducted in the Transonic Wind Tunnel Göttingen of the German–Dutch Wind Tunnels (DNW-TWG), which is a closed-circuit, variable density wind tunnel [87]. DNW-TWG features three exchangeable test sections, which enable subsonic to supersonic flow conditions to be covered in the same facility. The adaptive-wall test section, which allows the implementation of freestream Mach numbers from M = 0.3 to 0.9, was used in the present study. An image of the adaptive-wall test section with the mounted wind-tunnel model and experimental techniques is shown in Figure 1.

The freestream Mach number is evaluated from the measurements of the flow total pressure and freestream static pressure via the isentropic flow equation. The freestream Reynolds number is obtained from the freestream Mach number, the measurements of the flow total pressure and total temperature, and the freestream dynamic viscosity, which is determined according to Sutherland’s law. The freestream static temperature, which is necessary for the determination of the dynamic viscosity, is evaluated from the freestream Mach number and the measured flow total temperature via the isentropic flow equation. Details on the wind-tunnel data acquisition system, on the procedures for the evaluation of the freestream parameters, and on the related measurement uncertainties are reported in [48]. In the present work, the freestream Mach and Reynolds numbers were kept constant (within a tolerance of ΔM = ±0.003 and ΔRe = ±0.05 × 10^6^) around the set point values of M = 0.72 and Re = 9.7 × 10^6^, respectively, where the Reynolds number is based on the model chord length *c* = 1 m.

The upper and lower test-section walls are adaptive and enable, in general, interference-free contours to be set [48,88,89]. However, at the test conditions examined in this work, with an angle of attack of AoA = 1.5° (accuracy of ±0.016°), the adaptation of the test-section walls could not converge, because the local flow velocity in the proximity of the upper and lower walls was outside of the application limits of the wall adaptation algorithm [48,88,89]. This was due to the size of the considered model, which was significantly larger than that of airfoil models typically investigated in the adaptive-wall test section of the DNW-TWG (typical model chord length *c* = 0.3 m [89]). Nevertheless, pre-defined model contours, identified in a preliminary investigation [48], were set in the present experiments, thus allowing for the repeatability and reproducibility of the experimental results. At this point, it should be emphasized that the examined experimental configuration with a large airfoil model was selected as an optimal condition for the development and validation of measurement techniques, because it enabled investigations at high chord Reynolds numbers with a large measurement surface [48]. In fact, the focus of these experiments was not the evaluation of the aerodynamic performance of an airfoil. The main reasons for the installation of the wake rake shown in Figure 1 were also the repeatability and reproducibility of the flow conditions considered in other experiments [48], since its position (420 mm downstream from the airfoil trailing edge) was too close to the model to measure the airfoil drag correctly [90]. Therefore, only an estimation of the airfoil drag coefficient (via wake-deficit integration) was possible.

### 3.2. Wind-Tunnel Model and Measurement Techniques

**Figure 2:**
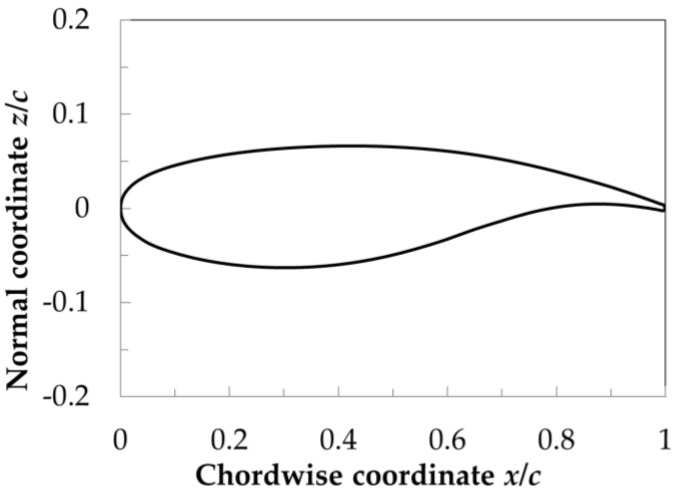
VA-2 supercritical airfoil [91,92]. The axes are not equally scaled.

The examined two-dimensional model had, as a cross-section, the VA-2 supercritical airfoil [91,92] shown in Figure 2. In this work, *x* is the chordwise coordinate, positive from the model leading edge to the model trailing edge; *y* is the spanwise coordinate, positive from the model port side to the model starboard side; and *z* is the coordinate perpendicular to the model surface, positive upward. The VA-2 supercritical airfoil was designed for a small change in the shock-wave location at off-design conditions, and was investigated in various studies (see [48,61,62,91,92,93], among others). It should be emphasized here that most of the previous investigations focused on turbulent boundary layers, except for [48,62], in which free transition was examined. In [62], however, the investigated range of Mach numbers was below M = 0.72, which was the Mach number considered in [48] and in the present study.

**Figure 3:**
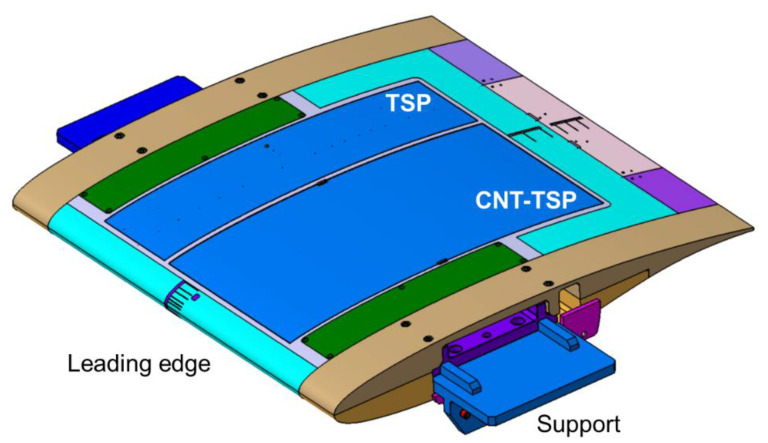
Construction drawing of the VA-2 supercritical airfoil [94] with the CNT-insert (upper side). The different colors indicate the different model parts; see the main text.

The VA-2 airfoil model with a 1 m chord by a 1 m span used in [48,61,62] was adapted in this work for the application of the TSP measurement technique. A construction drawing of the model (upper side) is shown in Figure 3. As can be seen in this figure, the model was composed of different parts, which are indicated by different colors.

The main part of the model, shown in cyan, also comprised the model leading edge and the model lower side (the latter is not visible in the figure), and was connected to the trailing-edge part (violet/pink in Figure 3). The central part of the model upper side was an exchangeable insert (gray and blue in the figure), which extended in the streamwise direction over the region *x*/*c* = 4.7% to 81.8%. The remaining model components were the covers and the side parts (indicated in Figure 3 by the green and brown colors, respectively). The model was mounted on two supports (blue in the figure), which were fixed to turntables located at the lateral test-section walls of the DNW-TWG. All model parts were made of aluminum.

The main and trailing-edge parts of the model were equipped with a main row of 36 pressure taps, distributed along the mid-span model section (*y*/*c* = 0.5), in order to measure the surface pressure distribution. All pressure taps had a diameter of 0.3 mm. The model surface pressures were measured using electronic pressure-scanning modules with an accuracy of ±62 Pa.

Two different inserts were specifically designed and manufactured for the application of the TSP measurement technique. The main difference between the two inserts was the heating system integrated beneath the TSP, as will be explained in the following two subsections. The two inserts were investigated separately in two different phases of the experimental campaign (see Section 3.3).

#### 3.2.1. Model Insert with a CNT-Heating (CNT-Insert)

The TSP on the first model insert was applied in two pockets machined into the insert surface. The applied TSP layers completely filled the pockets, with no contour variations being present at the interfaces between the TSP areas and the metallic model surfaces. The regions where the TSP was applied are visible in Figure 1 (yellow regions) and are indicated by the blue areas in Figure 3. Both pockets extended from *x*/*c* = 5.8% to 80.7%.

The composition of the TSP layers in the port pocket was designed to integrate a layer of CNT, in a manner analogous to that presented in [65,70,72], but with the layer thicknesses adjusted for the considered transonic flow conditions at a high Reynolds number. In order to guarantee the adhesion of the paint to the metallic surface, the model was first coated with a primer layer; a white screening layer for thermal and electrical insulation was then applied on the primer layer, and the layer of CNT was applied on this first screening layer; a second screening layer, with the same properties as the first one, was applied on the CNT layer (also functioning as a diffusive light-scattering background); finally, the active layer, in which the luminophores were embedded, was applied on the screening layer. The final thickness of the five-layer CNT-TSP in the port pocket was 450 μm. The electrical connections for the CNT were copper strips applied on the first screening layer before the application of the CNT layer. They were oriented in the streamwise direction and located at the spanwise ends of the pocket. The wires used to supply the electrical power to the CNT from outside of the test section were routed through the model inner volume to the power supply. Since the electrical connections for the CNT were covered by the upper TSP layers, neither they nor the electrical wires affected the final model surface quality. The electrical resistance of the CNT system, wires included, was approximately 110 Ω.

The TSP in the starboard pocket had no integrated heating. It served as a reference for the evaluation of the adiabatic-wall temperature distribution from the TSP intensity distribution (see Section 2.2.1 and Section 4). The composition of the TSP layers in this pocket was analogous to that typically used in wind-tunnel testing [64,69], i.e., with a primer, a screening and an active layer. The final thickness of the three-layer TSP in the starboard pocket was 180 μm.

The components of the used TSP (primer, screening, and active layers) were the same for both TSP regions. In particular, the TSP active layer consisted of a europium complex (luminophore) incorporated in a commercial polyurethane clear-coat binder [95]. The luminophore is excited in the wavelength range *λ*_ex_ = 350–450 nm, and it emits in the wavelength range *λ*_em_ = 600–630 nm. The luminescent lifetime of the used TSP is of the order of 200–300 µs in the temperature range 295–315 K [95]. The CNT was mixed into the same polyurethane binder material as that in which the luminophores were incorporated [65,72]

Black markers with a circular shape were applied in both TSP regions to the surface of the screening layer located below the active layer. They were used for TSP image preprocessing (see Section 4.2 and [5,69]). Note that the markers were applied before the model had been coated with the active layer, and therefore had no influence on the final model surface quality.

The starboard area of the insert was also instrumented with 15 pressure taps to measure the surface pressure distribution over the insert. The pressure taps were distributed in the chordwise direction, and were located at *y*/*c* = 0.68. Analogously to the pressure taps on the main and trailing-edge parts of the model, the pressure taps on the insert also had an orifice diameter of 0.3 mm, and the surface pressure distribution was acquired by means of the same measurement system used for the model pressures. The majority of the insert pressure taps (14 of 15) were embedded in the starboard TSP region. The quality of the pressure taps (circular shape of the orifices, tap diameter, and sharpness of the orifice edges) was ensured for all pressure taps via an additional treatment of the orifices after the TSP application, as described in [70]. The spanwise position of the pressure taps was selected at *y*/*c* = 0.68, instead of *y*/*c* = 0.5, in order to avoid the risk of damaging the CNT layer in the port TSP region. By combining the surface pressures measured at *y*/*c* = 0.5 with those measured at *y*/*c* = 0.68, the whole surface pressure distribution over the airfoil could be determined. The combination of the surface pressures measured at two different spanwise locations to obtain the airfoil pressure distribution was allowable, since the pressure distributions measured at these two spanwise locations had already been verified to agree in earlier work [48].

Seven fast-reacting thermocouples (type K, wire diameter 0.08 mm, accuracy ±0.3 K) were additionally installed in the TSP starboard area. The thermocouples were positioned at different chordwise locations and embedded in orifices with a diameter of 1 mm. Six of the thermocouples were mounted at *y*/*c* = 0.739 in such a way that their junction end was situated within the active layer of the TSP coating, thereby enabling an accurate measurement of the surface temperature. The remaining thermocouple was mounted at *y*/*c* = 0.719, 1 mm below the model surface, in order to compare the temperature measured here with the values measured at the surface.

#### 3.2.2. Model Insert with CFRP Heating (CFRP-Insert)

**Figure 4:**
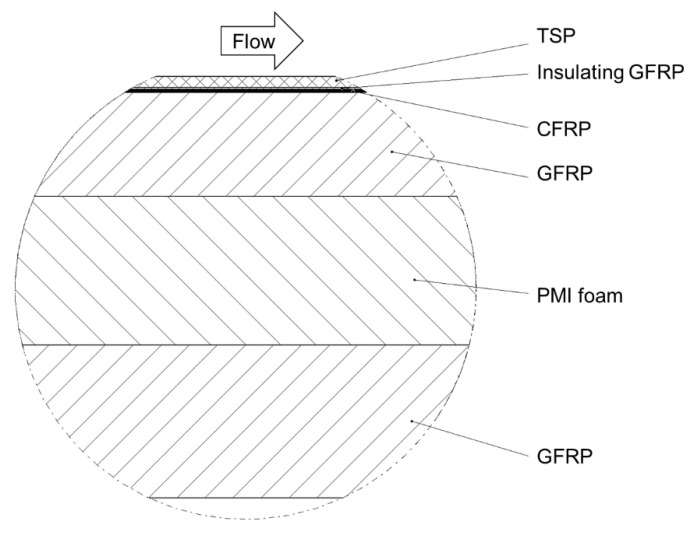
Sketch of the layer design of the laminate manufactured for the CFRP-insert [96].

The second insert consisted of two main components: a laminate manufactured of fiber-reinforced plastic, and a frame structure made of aluminum. The design of the laminate was based on that presented in [74] and further developed for TSP applications in [73], but was optimized for the examined flow conditions, at which a high aerodynamic loading acted on the model. The layer design of the present laminate is sketched in Figure 4 and described below from the top to the bottom layer, as seen from the model surface [96]. The application of the different layers also followed this order.

The laminate was manufactured in a negative mold made of aluminum, on which a release agent was applied. After curing, the TSP active layer (with the same formulation of that described in Section 3.2.1, and a thickness of approximately 50 µm) was applied to the surface of the release agent. A thin layer (80 µm) of Glass-Fiber-Reinforced Plastic (GFRP) was placed onto the TSP in order to guarantee electrical insulation. The current-carrying carbon fiber layer (Carbon-Fiber-Reinforced Plastic, CFRP), with a thickness of 100 µm, was then applied. The further three, thicker layers carried most of the structural loading. They were two GFRP layers and a polymethacrylimide (PMI) foam layer. The total thickness of the laminate was 15 mm. In order to minimize the deformation of the insert under aerodynamic loading, the laminate was then connected to an aluminum frame structure [96]. Similarly to those used for the CNT layer, the electrical connections for the CFRP layer were also copper strips, applied at the spanwise ends of the CFRP layer and oriented in the streamwise direction. The connection of electrical wires was accomplished by riveting threaded copper bars to the copper strips, so that the wires could be connected via screws after the insert assembly. The electrical resistance of the CFRP system, wires included, was approximately 0.7 Ω, i.e., markedly lower than that of the CNT system (see Section 3.2.1).

**Figure 5:**
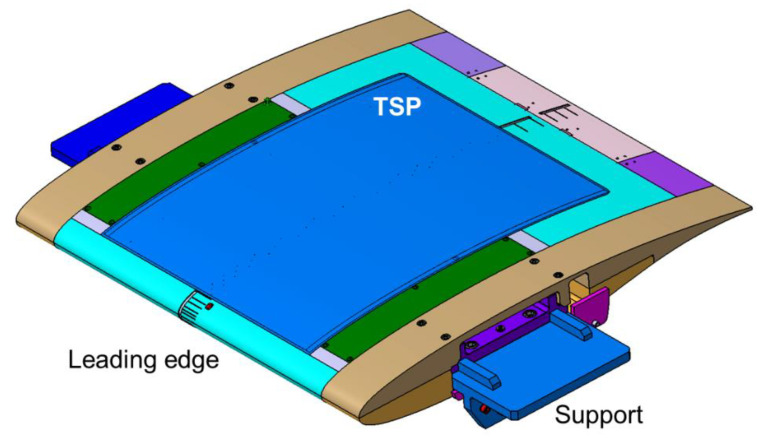
Construction drawing of the VA-2 supercritical airfoil [94] with the CFRP-insert (upper side). The different colors indicate the different model parts.

**Figure 6:**
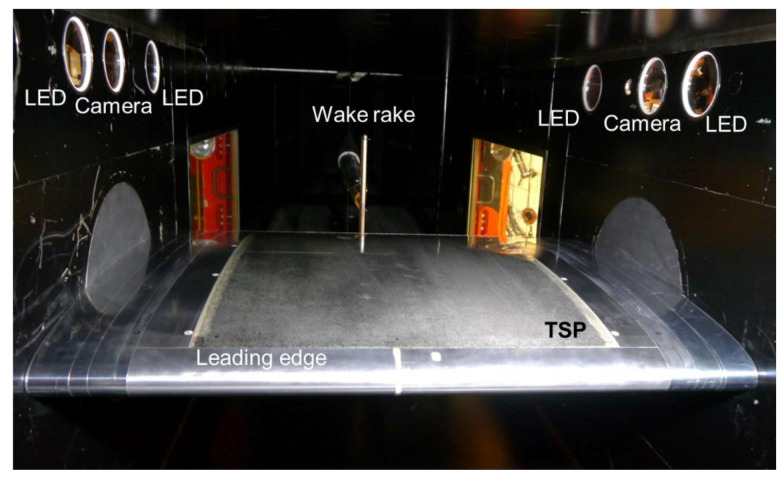
VA-2 supercritical airfoil model with the CFRP-insert and measurement techniques installed in the DNW-TWG test section. LEDs and cameras are behind the indicated windows. Room light on, LEDs off.

A construction drawing of the model (upper side) with the CFRP-insert is shown in Figure 5, in which the different components are indicated by the same colors as were used in Figure 3. As can be clearly seen in Figure 5, the TSP area almost covered the whole surface of the CFRP-insert. In this case, the TSP extended from the front to the rear insert-metal interface, i.e., from *x*/*c* = 4.7% to 81.8%. An image of the wind-tunnel model with the CFRP-insert mounted in the adaptive-wall test section of the DNW-TWG is presented in Figure 6. In this figure, the TSP appears to be significantly darker than that on the CNT-insert (see Figure 1). This was due to the absence of a screening layer between the TSP active layer and the CFRP heating layer.

Black markers with a circular shape were applied onto the TSP surface after the insert manufacturing. Therefore, the markers were, in this case, exposed to the flow. Nevertheless, their height (approximately 5 µm) was sufficiently small with respect to the boundary-layer thickness, so that the (weak) disturbances induced by the markers did not lead to the formation of turbulent wedges.

Additionally, the CFRP-insert was instrumented with 35 pressure taps in order to measure the surface pressure distribution, and with 6 thermocouples to monitor the laminate temperature below the TSP. The holes for these additional sensors were drilled through the laminate before connecting it to the aluminum frame structure. The characteristics of pressure taps and thermocouples were the same as those installed in the CNT-insert. The pressure taps were installed in the mid-span model area (within the region 0.49 ≤ *y*/*c* ≤ 0.51), distributed in the chordwise direction at the same locations as those used in [48]. The thermocouples were placed at the same chordwise and spanwise locations as the six thermocouples in the CNT-insert that were embedded in the TSP layer, but in this case the holes for the thermocouples ended at a distance of 0.3 mm from the TSP surface. The junction ends of the thermocouples were fixed in the proximity of the ends of these holes. Therefore, all thermocouples of the CFRP-insert were positioned below the TSP layer, i.e., in the second GFRP layer but very close to the CFRP layer.

### 3.3. Optical Setup

Because optical access perpendicular to the model upper side was not possible due to the adaptive lower and upper walls [48], the TSP hardware had to be installed behind the side walls of the test section. The experimental campaign was subdivided into two main phases, with different optical setups:

- In the first phase of the test campaign, one high-speed camera was used to investigate the model with the CNT-insert. This camera was a Complementary Metal-Oxide-Semiconductor (CMOS) Photron FASTCAM Mini AX200 camera, which has a 12-bit dynamic range and was operated with a 1024 × 672 pixels image sensor. The CMOS camera was equipped with a 24 mm focal length lens and mounted behind one of the circular windows at the starboard test-section wall (see Figure 1). A band-pass filter for the wavelength range of 590–670 nm was mounted in front of the camera lens, thus allowing the light emitted by the TSP to be captured while at the same time blocking light at shorter and higher wavelengths. During this first phase of the experimental campaign, TSP images were acquired at *f*_acq_ = 1 kHz (the camera shutter speed was 1/frame s).
- In the second phase of the test campaign, two Charge-Coupled Device (CCD) pco.4000 cameras were used to investigate the model with the CFRP-insert. They have a 14-bit dynamic range and high spatial resolution (4008 × 2672 pixels image sensor), but also have a relatively low frame rate: in this phase of the experimental campaign, the TSP images were acquired at *f*_acq_ = 3.3 Hz (CCD exposure time of 90 ms). Each camera was equipped with a 24 mm focal length lens, and band-pass spectral filters for the wavelength range of 600–700 nm were mounted between the camera lenses and the CCD chips. One camera was mounted at each test-section side, as shown in Figure 6.

In both test-campaign phases, two LEDs were installed at each test-section side behind circular windows (see Figure 1 and Figure 6). The LEDs were HARDsoft IL-105/6X Illuminators with an excitation center wavelength of 390 nm. Band-pass filters, blocking light at λ < 350 nm and λ > 420 nm, were placed in front of the LEDs in order to avoid reflections of the illumination on the model surface.

## 4. TSP Data Acquisition and Processing

### 4.1. TSP Data Acquisition

The TSP data acquisition procedure was basically the same for both test-campaign phases. Before the wind-tunnel operation, a set of TSP images (named “wind-off”) were acquired at quiescent conditions, with the model set at the desired angle of attack (AoA = 1.5°) and the air pressure set equal to the flow total pressure during the runs (*p*_0_ = 80 kPa). At these quiescent conditions, the model surface temperature was reasonably assumed to be uniform, since the differences between the temperatures measured by the installed thermocouples were within their accuracy (typically within Δ*T* = ±0.15 K). The wind-off TSP images served as calibration data for the determination of the surface temperature distribution from the TSP images acquired during the wind-tunnel operation, as discussed below.

As soon as the desired flow conditions (M = 0.72 and Re = 9.7 × 10^6^) had been reached and had become stable, a set of TSP reference images (named “Ref”) was acquired. The heating system was then activated, and a set of TSP images with heated surface (named “Run”) was acquired after 5 s of the heating system activation.

The number of TSP images acquired for the three image sets was obviously different for the two test-campaign phases. In the first phase of the experimental campaign, in which the high-speed camera was used, 513 TSP images were recorded at the wind-off and Ref conditions, whereas 4097 TSP images were recorded at the Run conditions. In the second phase of the experimental campaign, 20 TSP images were recorded for each set of images.

The electrical power applied to the CNT layer in the first phase of the test campaign was 800 W/m^2^, whereas the electrical power applied to the CFRP layer in the second phase of the test campaign was 860 W/m^2^.

### 4.2. Preprocessing of the TSP Images

The preprocessing of the TSP images was performed by means of the DLR software *ToPas* [97]. The initial phase of the TSP image preprocessing was carried out in the image plane. The wind-off and Ref images were first averaged to improve the signal-to-noise ratio. Under the flow conditions, the VA-2 model slightly deformed because of the effect of the aerodynamic loading. Consequently, the luminescent model image in the Ref and Run images was also deformed with respect to that in the wind-off images (no aerodynamic loading). The black circular markers applied to the model surface (see Section 3.2) were used to align the Ref and Run images to the wind-off images (image registration), following the procedure described in [5]. After the image registration had been accomplished, the wind-off images were divided by the Ref and Run images. The divided images were essential for the calibration of the TSP images to obtain the surface temperature distribution. Prior to TSP data calibration, however, the divided TSP images were projected (mapped) onto a three-dimensional grid representing the model upper surface. The considered model surface was different for the two phases of the experimental campaign. In the first phase of the test campaign, only a part of the TSP surface could be captured by the high-speed camera. Therefore, the considered portion of the model surface represented by the three-dimensional grid extended from *x*/*c* = 6.0% to 43.5% for almost the whole span width of the TSP port region of the CNT-insert (*y*/*c* = 0.235 to 0.56). In the second phase of the test campaign, each camera could observe the whole opposite half of the TSP surface. Essentially the full TSP area of the CFRP-insert (*x*/*c* = 6.0% to 80.3% for *y*/*c* = 0.216 to 0.784) was hence considered for the three-dimensional grid. In fact, the divided TSP images from the two cameras were merged at the mid-span model section, leading to one combined image over the whole examined surface. The considered portions of the insert surface were discretized using structured grids, both of which had the same resolution of Δ(*x*/*c*) = Δ(*y*/*c*) = 0.001. The mapping of the divided TSP images was carried out according to the methodology described in [5,97], which was based on the positions of the aforementioned circular markers in the image plane and in the three-dimensional space.

**Figure 7:**
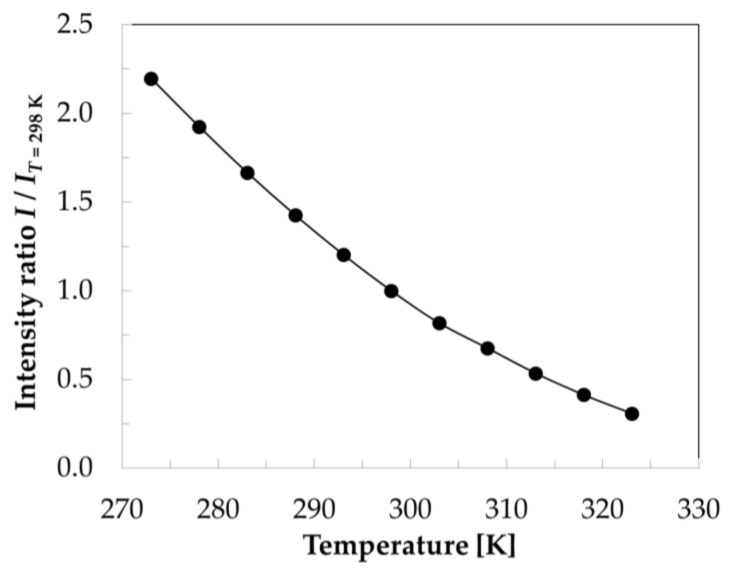
Calibration function for the used TSP [95].

The surface temperature distributions were evaluated quantitatively by applying a calibration function to the ratios of the TSP intensity distributions in the wind-off/Ref and wind-off/Run images, which had been mapped onto the three-dimensional grids. The calibration function between the TSP intensity ratio and the surface temperature was determined experimentally in [95], and is shown in Figure 7.

**Figure 8:**
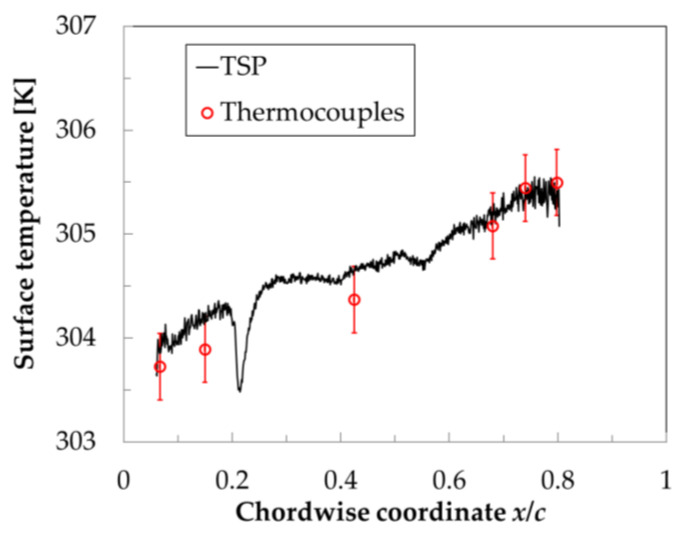
Comparison of the surface temperature measured via TSP and thermocouples on the starboard (i.e., unheated) TSP region at M = 0.72, Re = 9.7 × 10^6^ and AoA = 1.5° [98].

As discussed in Section 4.1, the model surface temperature in the wind-off images was reasonably assumed to be uniform; it was taken as the average of the temperatures measured by the thermocouples at those quiescent conditions. After the application of the calibration function to the mapped wind-off/Ref and wind-off/Run data, the surface temperature distributions were obtained for both the Ref and Run conditions. An example of a streamwise surface temperature distribution extracted from the TSP data at the Ref conditions is shown in Figure 8. These data were measured in a previous work [98] on the starboard TSP region of the CNT-insert, and allowed the surface temperatures obtained from the TSP to be compared with those measured by the thermocouples, which are also shown in Figure 8. The temperature profile was extracted from the TSP region at 0.70 ≤ *y*/*c* ≤ 0.75, i.e., by averaging the surface temperature in the spanwise direction over 50 mm about the location of the thermocouples (*y*/*c* = 0.739). Spanwise averaging was justified because the flow was essentially two-dimensional in the examined region [98]. It can be seen in Figure 8 that the surface temperature distribution obtained via TSP was in agreement with the temperatures measured by the thermocouples (the differences were within the measurement uncertainty of ±0.3 K). As expected for the examined compressible flow, the streamwise temperature distribution was non-uniform even for the unheated TSP surface (Ref conditions).

### 4.3. Spatial Filtering of the TSP Data

While the preprocessed TSP data fed the algorithms used in the TH and TR approaches without any further manipulation, the temperature maps used in the OF approach required preliminary spatial filtering. The adopted filter, described in [25], consisted of a modified gaussian blurring filter with the kernel slightly shrunk in the direction of the dominant local gradient. The undeformed gaussian support was 3 grid nodes in diameter; the maximum allowed shrinkage, extracted locally at every node, was given by the ratio between the eigenvalues of the correlation matrix of the spatial temperature gradients, and could not exceed 1.3. Its application minimized the white, additive, gaussian noise, while at the same time preserving the existing edges, i.e., the temperature gradients.

## 5. Results and Discussion

**Figure 9:**
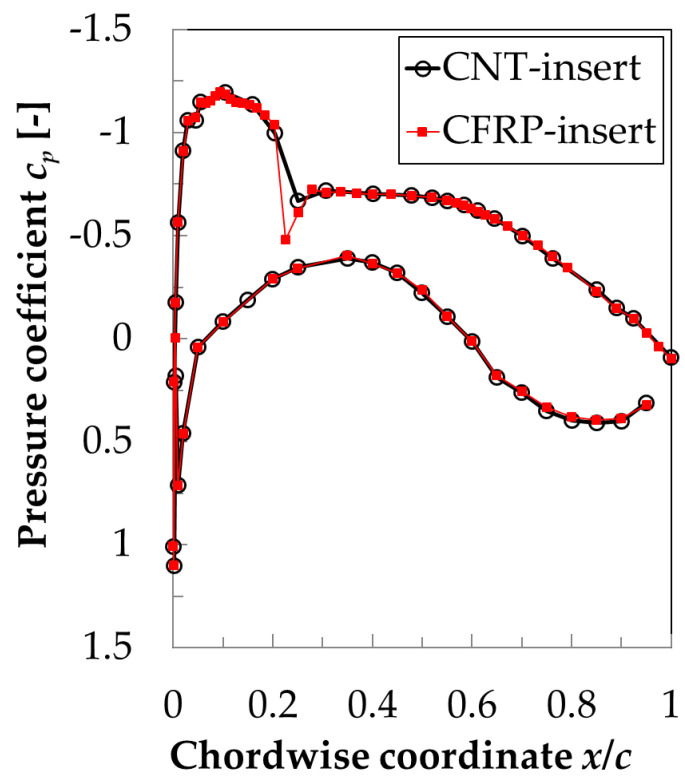
Comparison of the pressure distributions measured at M = 0.72, Re = 9.7 × 10^6^ and AoA = 1.5° on the VA-2 airfoil model with the two different inserts.

This work focused on transonic flow conditions at a high-chord Reynolds number. The surface pressure distributions measured at the examined test conditions (M = 0.72, Re = 9.7 × 10^6^ and AoA = 1.5°) in the two phases of the experimental campaign (i.e., with CNT-insert and with CFRP-insert) are shown in Figure 9. The curves on the top of the figure were obtained on the model upper side. After a strong acceleration over the leading-edge region up to locally supersonic flow conditions, the boundary layer underwent a deceleration at approximately *x*/*c* > 10%, culminating in a very strong adverse pressure gradient at approximately 20.5% ≤ *x*/*c* ≤ 22.5% related to a shock wave, which terminated the supersonic flow region. Further downstream, the boundary layer encountered a region of nearly-zero pressure gradient (up to *x*/*c* ~ 50%), followed by another region of adverse pressure gradient up to the trailing edge. As can be seen in Figure 9, the surface pressure distributions measured in the two phases of the test campaign were in excellent agreement on both the model upper and lower sides, except for small differences measured at approximately 15% ≤ *x*/*c* ≤ 25%. These small differences were likely related to small differences in the strength and position of the shock-wave between the runs, as well as between the spanwise locations of the pressure tap rows. It should be also noted here that the spatial resolution of the pressure tap rows on the CNT- and CFRP-inserts was different; in particular, a pressure tap at *x*/*c* = 21.6% was available only on the CFRP-insert. This aspect has to be kept in mind when the region between the pressure taps at *x*/*c* = 20.4% and 22.5% is considered; otherwise, the apparently different lines connecting the pressure coefficients measured in this region may be misinterpreted.

**Figure 10:**
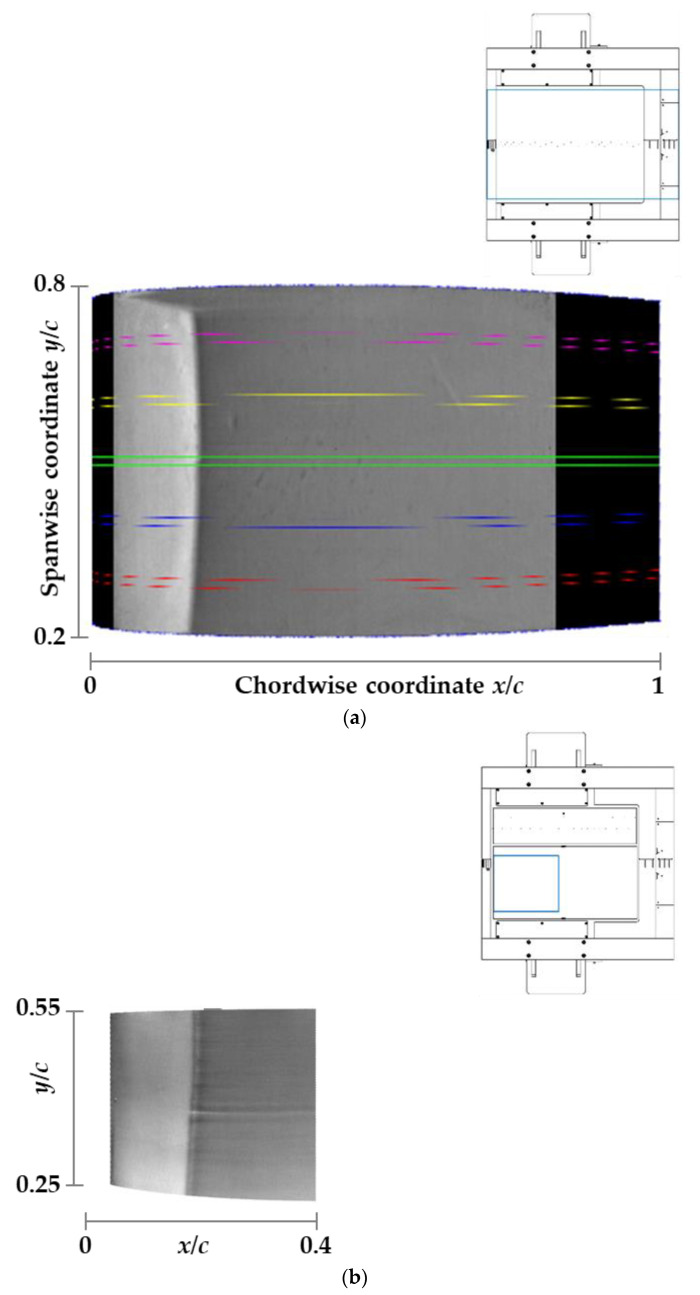
TSP results obtained at M = 0.72, Re = 9.7 × 10^6^ and AoA = 1.5° on the VA-2 airfoil model with the CFRP-insert (**a**) and the CNT-insert (**b**). The classical representation of the TSP results chosen for this figure visualizes regions of low and high wall heat flux (and therefore of low and high skin friction) as bright and dark areas, respectively. The examined areas are indicated by cyan rectangles in the engineering drawings shown above the corresponding TSP results.

The TSP results obtained for the examined test conditions with the CFRP- and CNT-inserts are presented in Figure 10a and Figure 10b, respectively. The flow is from the left; the bright and dark areas correspond to regions of low and high wall heat flux (and therefore of low and high skin friction). The results are shown as they would be seen from the (inaccessible) top wall of the DNW-TWG test section, with the observer located above the center of the model, looking perpendicularly to the model plane. This is a typical representation of a TSP result common to several other publications (see [5,65,69], among others). In Figure 10a, the whole model chord length for the spanwise region 0.216 ≤ *y*/*c* ≤ 0.784 is shown. The area examined on the CNT-insert was significantly smaller than that on the CFRP-insert, as can be seen by a comparison of Figure 10a and Figure 10b. The presentation of the results obtained with the CNT-insert is limited to the area between *x*/*c* = 6% and 40% for the spanwise region between *y*/*c* = 0.25 and 0.54. The examined areas are indicated by cyan rectangles in the engineering drawings shown above the corresponding TSP results in Figure 10.

A strong variation of the wall heat flux (and therefore of the skin friction) occurring in the streamwise direction at approximately *x*/*c* = 21% can clearly be seen in Figure 10. It was due to the shock-induced transition of the boundary layer from the laminar to the turbulent state, which led to a marked increase of the skin friction in the streamwise direction. On the other hand, the transition front was essentially two-dimensional for most of the considered span width, appearing as a nearly straight line in the spanwise direction, with a slight upstream “bending” in the regions closer to the spanwise ends of the TSP area. This behavior can be especially appreciated in Figure 10a, since the spanwise extent of the examined TSP region was larger than that in Figure 10b. For this reason, the following discussion relies on the TSP result presented in Figure 10a.

The turbulent wedge arising at the starboard end of the TSP region was very likely caused by a small spanwise discontinuity in the insert contour at the TSP–metal interface, which size was, however, relatively large with respect to the thickness of the laminar boundary layer at this upstream location. This type of contour discontinuity is expected to induce three-dimensional disturbances, which would lead to the formation of a turbulent wedge, such as that observed in Figure 10a. The colored lines in the figure show the bounds of five evaluation sections located at *y*/*c* = 0.3, 0.4, 0.5, 0.6 and 0.7, along which the location of the boundary-layer transition was determined at the position of the maximal streamwise temperature gradient; this analysis was accomplished by means of the algorithm described in [99]. Over the considered spanwise region of Δ(*y*/*c*) = 0.4, the variation of the transition location was very small: from *x*_T_/*c* = 20.4% to 21.3%.

The following sections will focus on the results of the analysis of the TSP data to detect the critical lines on the CNT and CFRP-inserts, based on the approaches presented in Section 2.2.

### 5.1. Topology of the Skin-Friction Lines Obtained via the OF Approach

**Figure 11:**
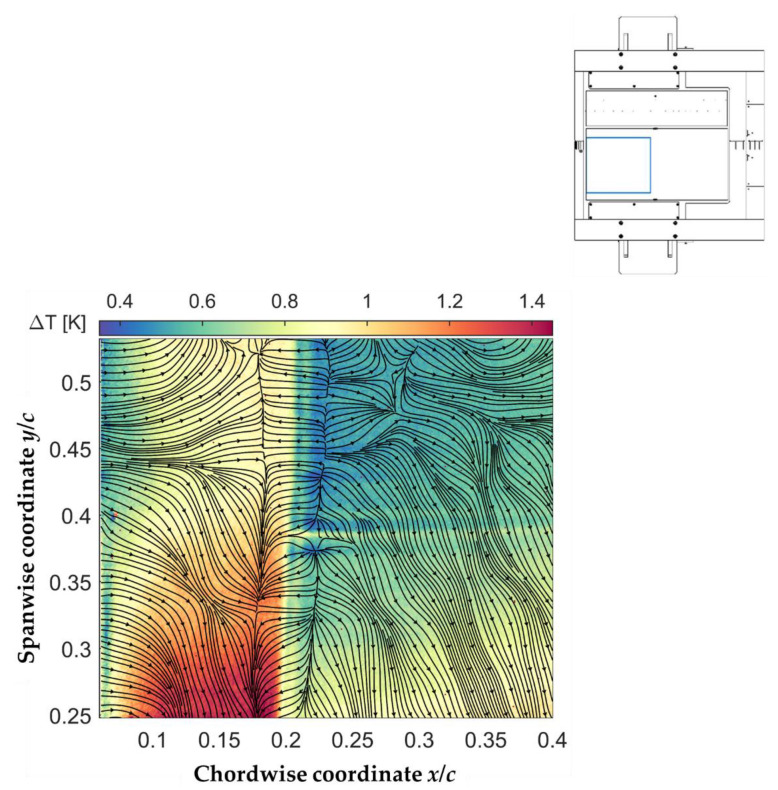
Temperature difference map Δ*T* = (*T_w_*(*x*,*y*) − *T_ref_*(*x*,*y*)) with superimposed skin-friction lines obtained via the OF approach on the VA-2 airfoil model with the CNT-insert, investigated at M = 0.72, Re = 9.7 × 10^6^ and AoA = 1.5°. The top figure shows the examined area, indicated by a cyan rectangle in the engineering drawing.

As discussed in Section 2.2.1, the OF approach was applied to the temperature difference map Δ*T* = (*T_w_*(*x*,*y*)−*T_ref_*(*x*,*y*)) with, in this case (see Section 4):

- *T_w_*(*x*,*y*) = *T*_*Run*,*avg*_(*x*,*y*);
- *T_ref_*(*x*,*y*) = *T_aw_*(*x*,*y*) = *T*_*Ref*,*avg*_(*x*,*y*),

where the subscript “avg” indicates the average of the corresponding TSP dataset, i.e., the time-averaged data. The surface temperature distribution with heated surface *T_w_*(*x*,*y*) was also obtained from the average of the Run TSP data, in a manner similar to that of the *T_ref_*(*x*,*y*) distribution (which was obtained from the average of the Ref TSP data). The temperature difference map obtained at the examined test conditions in the first phase of the test campaign (CNT-insert investigated with the high-speed camera) is shown in Figure 11. In this and in the following figures, the representation of the results is slightly different from that of Figure 10, since they are shown in the *x*–*y* plane. The used color map emphasizes a major issue affecting the temperature difference map shown in Figure 11: the temperature increase induced by the CNT-heating was non-uniform in the spanwise direction. In particular, the region at approximately *y*/*c* < 0.4 seemed to be heated more than the region at approximately *y*/*c* > 0.4. Nevertheless, significant changes were observed in the streamwise distribution of the temperature difference (*T_w_*−*T_ref_*), especially in the region around the shock-induced transition location (approximately 17% < *x*/*c* < 24%). The OF approach was applied to this temperature map in order to determine the skin-friction lines, which are also presented in Figure 11 (superimposed over the temperature difference map). In this, and in the following figure, the skin-friction distribution was evaluated with the lagrangian multiplier [24,56] *α* = 10, and with *η* = 1000 (see Section 2.2.1). This latter value is the same as that chosen in another work [56], while the value of *α* was selected after an iterative process.

A glance at Figure 11 clearly shows that the flow-independent temperature gradients (i.e., those induced by the non-uniform CNT-heating) had a strong impact on the skin-friction fields resulting from the OF approach, especially in the turbulent region (at approximately *x*/*c* > 24%). In fact, the temperature gradients in the spanwise direction induced unphysical spanwise-oriented skin-friction lines; these were particularly pronounced in the turbulent region, where the temperature difference (*T_w_*−*T_ref_*) was small and flow-independent effects became predominant. In spite of this, the skin-friction lines were clearly shown to converge and diverge at a *x*_S_/*c* ~ 18% and *x*_R_/*c* ~ 22%, respectively. As introduced in Section 2.2.1, the locations of converging and diverging skin-friction lines correspond to the flow separation and reattachment locations. A laminar separation bubble was thus detected in the region at approximately 17.5% < *x*/*c* < 23.5%. The occurrence of an LSB in this region is in line with the expectations for the considered SWBLI at the examined test conditions (see Section 1). The detected locations of separation and reattachment were in reasonable agreement with the upstream and downstream ends of the region of marked adverse pressure gradient related to the shock (see Figure 9). A quantitative comparison of the current results with reference data is presented in Section 5.3. The variations of the separation and reattachment locations over the considered span width were approximately Δ(*x*_S_/*c*) = ±0.6% and Δ(*x*_R_/*c*) = ±0.9%, respectively. As shown also in Figure 10, this slight spanwise variation was not related to the flow-independent temperature gradients, but rather a result of the three-dimensional SWBLI in the regions closer to the test-section side walls. This slight spanwise “bending” of a nearly two-dimensional laminar separation bubble will also be confirmed by the following results, obtained with the CFRP-insert.

**Figure 12:**
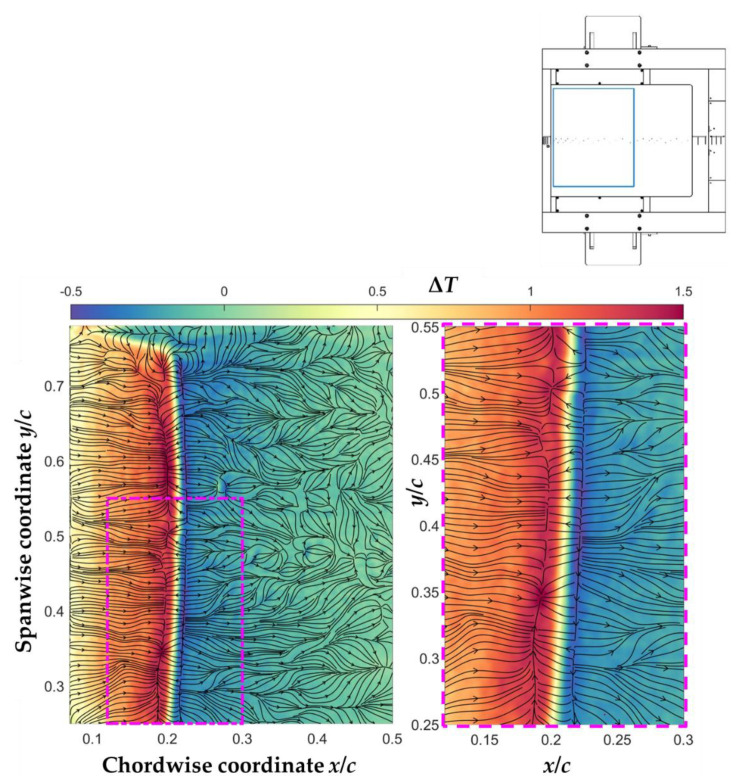
Temperature difference map Δ*T* = (*T_w_*(*x*,*y*)−*T_ref_*(*x*,*y*)) with superimposed skin-friction lines obtained via the OF approach on the VA-2 airfoil model with the CFRP-insert, investigated at M = 0.72, Re = 9.7 × 10^6^ and AoA = 1.5°. The left figure shows the results in the whole examined area, which is indicated by a cyan rectangle in the engineering drawing presented in the top figure. The right figure shows a zoomed-in presentation of the temperature difference map and of the skin-friction lines in the magenta rectangle indicated in the left figure.

It can be already seen in Figure 10 that the TSP data measured with the CFRP-insert (second phase of the test campaign, using two CCD cameras) were less affected by flow-independent temperature gradients. This is more clearly visible in Figure 12, in which the results are presented in the same manner as in Figure 11. Almost the whole span width of the TSP area on the CFRP-insert is shown in Figure 12 (left), with the presentation limited to *x*/*c* = 50% in the streamwise direction. The considered area is obviously larger than that shown in Figure 11, the spanwise extent of which essentially corresponds to that of the region enclosed in Figure 12 (left) by the magenta rectangle. A zoomed-in presentation of the temperature difference map (*T_w_*(*x*,*y*)–*T_ref_*(*x*,*y*)) and of the skin-friction lines in the region delimited by the magenta rectangle is shown in Figure 12 (right).

As clearly shown in this figure, the skin-friction lines in the region up to *x*/*c* ~ 30% were essentially oriented in the streamwise direction, since the CFRP heating provided a more homogeneous heat flux than the CNT heating. This guaranteed a negligible influence of the flow-independent temperature gradients on the Run TSP data, as compared to the flow-induced temperature gradients, thus allowing for the distinct identification of the converging and diverging locations of the skin-friction lines over the whole considered spanwise region. The spanwise-averaged separation and reattachment locations were found in Figure 12 (left) at *x*_S_/*c* = 19.5% and *x*_R_/*c* = 22.5%, with a variation over the span of approximately Δ(*x*_S_/*c*) = ±0.5% and Δ(*x*_R_/*c*) = ±0.5%. It should be noted here that the spanwise-averaged transition location, detected from the TSP result shown in Figure 10a, was found at *x*_T_/*c* = 21.0%, i.e., in the middle of the region enclosed by the separation and reattachment lines.

The small differences in the locations of the critical lines between Figure 11 and Figure 12, in particular of the separation line, were due to the small difference in the SWBLI between the two phases of the test campaign, as discussed above with regard to the pressure distributions shown in Figure 9. This will be quantitatively confirmed by the comparison with reference data presented in Section 5.3. The OF algorithm was thus shown to be robust with regard to the identification of the flow separation and reattachment lines, in spite of the artifacts affecting the TSP data, especially in the case of the measurements conducted with the CNT-insert.

In the turbulent region, flow-independent temperature variations did affect the skin-friction line topology obtained with the CFRP-insert. This effect was observed at approximately *x*/*c* > 30%. In this region, the streamwise pressure gradient was nearly zero or moderately adverse (see Figure 9), leading to a weakening of the temperature gradient in the streamwise direction. Moreover, the signal-to-noise ratio decreased, as compared to the laminar region, because the surface temperature increase above the adiabatic-wall level was smaller in the turbulent region. For these reasons, inhomogeneities in the distribution of the imposed heat flux led to appreciable inhomogeneities in the surface temperature distribution at approximately *x*/*c* > 30%. In practice, in the absence of a screening layer (which was not applied on the CFRP-insert, see Section 3.2.2), the texture of the CFRP heating layer became visible through the semi-transparent TSP active layer (see Figure 6); the thermal signature due to this texture imprinted on the TSP data, eventually affecting the topology of the skin-friction lines.

It is interesting to note, in Figure 11 and Figure 12, that the skin-friction lines between the reattachment line and *x*/*c* ~ 30% were partially arranged in a set of streamwise-oriented converging and diverging lines, which resemble those observed, e.g., on a circular cylinder in crossflow [24], where they were associated to streamwise-oriented, Görtler-like vortices. Such vortical structures were also observed in SWBLI cases (see, e.g., [9,100]), where they can be initiated by the streamline curvature in the separated boundary layer. In particular, their signature in SSLC data [9] was similar to the thermal signature observed in Figure 10b. Streamwise-oriented streaks were also found in other SWBLI studies (see [10,16], among others). Dedicated experiments would be needed to confirm the presence of such coherent flow structures, and to understand the mechanism causing the streamwise-oriented converging/diverging skin-friction lines observed in Figure 11 and Figure 12. The topology of the skin-friction lines within and around the turbulent wedge located at the starboard end of the TSP area in Figure 12 (left) is also worth of mentioning. It appears as a streamwise-oriented diverging line in the middle of the turbulent wedge, connected to converging lines outside of the wedge. Although this description seems reasonable in view of the flow structures developing in the presence of a turbulent wedge, the orientation of the skin-friction lines connecting the diverging and converging lines may be affected by the heating configuration. These spanwise-oriented skin-friction lines may be unphysical, and necessitate further investigation in a dedicated study.

### 5.2. Distributions of $U_{T}$ Obtained via the TH and TR Approaches

For the application of the two approaches based on the propagation celerity of temperature perturbations (see Section 2.2.2), maps of the traces of the temperature fluctuations at the wall ($T_{w}^{\prime }\left(x,y\right)$) were required. These maps were obtained from the time-resolved TSP data recorded in the first phase of the test campaign by subtracting *T*_*Run*,*avg*_(*x*,*y*), i.e., the average Run TSP data, from each single *T*_*Run*_(*x*,*y*) map. The Ref TSP data were indeed not required for the application of the TH and TR approaches.

#### 5.2.1. Distributions of $\left|U_{T}\right|$ Obtained via the TH Approach

**Figure 13:**
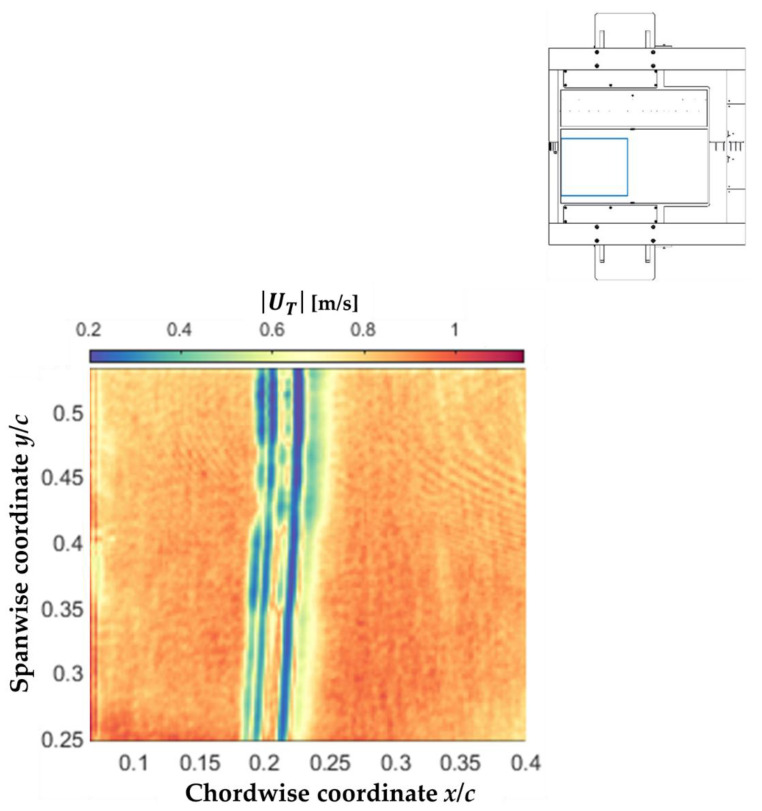
Map of the modulus of the streamwise component of the propagation celerity of temperature perturbations $\left|U_{T}\left(x,y\right)\right|$ obtained via the TH approach on the VA-2 airfoil model with the CNT-insert, investigated at M = 0.72, Re = 9.7 × 10^6^ and AoA = 1.5°. The top figure shows the examined area, indicated by a cyan rectangle in the engineering drawing.

The time-averaged distribution of the modulus of the streamwise component of ${\vec{U}}_{T}$, i.e., $\left|U_{T}\left(x,y\right)\right|$, was determined from the analysis of the whole set of $T_{w}^{\prime }\left(x,y\right)$ maps via the TH approach. The result obtained for the examined case at M = 0.72, Re = 9.7 × 10^6^ and AoA = 1.5° is presented in Figure 13, which shows the same TSP area as in Figure 11. Nearly straight lines of small $\left|U_{T}\right|$, essentially oriented in the spanwise direction, can be seen in the region at approximately 18% < *x*/*c* < 23%. This region was analyzed by looking at the minima of Equation (5), thus identifying the separation and reattachment locations. The downstream critical location, corresponding to the spanwise-averaged reattachment location, was found at *x*_R_/*c* = 22%, with a variation over the span of Δ(*x*_R_/*c*) = ±0.7%. This location of *x*_R_/*c*, with the relative variation over the span, was in significant agreement with that obtained via the OF algorithm (Figure 11). Two critical locations were found in the separation region, with spanwise averages at *x*_S_/*c* = 19% and 20%, and a similar variation over the span (Δ(*x*_S_/*c*) = ±0.7%). The upstream critical location was in reasonable agreement with the spanwise-averaged *x*_S_/*c* from the OF approach, and was thus identified as the separation location obtained via the TH approach. The second minimum of $\left|U_{T}\right|$ may indicate the presence of a secondary recirculating region within the (primary) separation bubble, such as that reported in [101]. This aspect will be investigated in detail in a future study. In this context, it should be noted that the analysis of the temporal evolution of the surface temperature data did not show indications of appreciable shock unsteadiness at the examined test conditions.

The TH approach was confirmed here to be only marginally exposed to flow-independent temperature gradients (see discussion in [57,75]). In fact, the distribution of $\left|U_{T}\right|$ was essentially uniform in the spanwise direction, as expected for the considered quasi-two-dimensional flow, with the only exception being the region around *y*/*c* = 0.25. This region was closer to the port electrical connection for the CNT-heating layer, and was significantly affected by a non-uniform temperature distribution (see Figure 10b and Figure 11), which might vary also in time, thus affecting the $\left|U_{T}\right|$ distribution determined via the TH approach. The origin of such flow-independent temperature fluctuations is currently unclear, and will be further investigated in future works.

Far from the critical lines, the determined propagation celerity of temperature perturbations seemed to saturate to a value around $\left|U_{T}\right|$ = 1 m/s. This effect was likely due to the still relatively low TSP image acquisition frequency, which did not enable the displacement of the thermal traces in the high-speed, attached flow regions to be captured via the TH approach. On the other hand, the friction velocity (and hence also $\left|U_{T}\right|$) obviously approached zero when the boundary layer underwent separation and reattachment. Therefore, the determined values of the propagation celerity of the temperature perturbations are expected to be correct in the vicinity of the critical lines.

#### 5.2.2. Distributions of $U_{T}$ Obtained via the TR Approach

**Figure 14:**
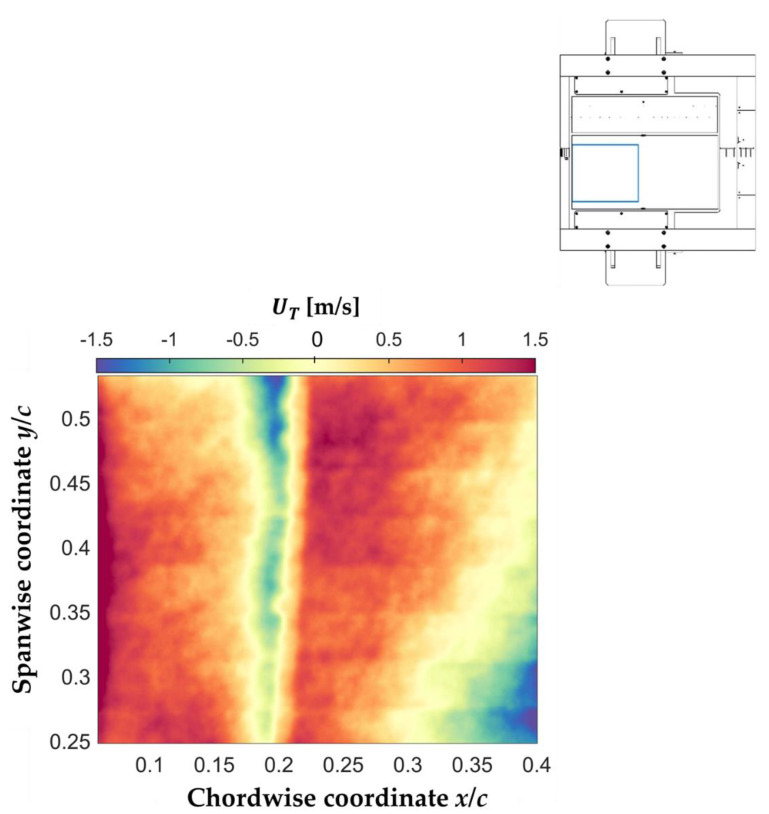
Map of the streamwise component of the propagation celerity of temperature perturbations $U_{T}\left(x,y\right)$ obtained via the TR approach on the VA-2 airfoil model with the CNT-insert, investigated at M = 0.72, Re = 9.7 × 10^6^ and AoA = 1.5°. Note that the color map used here for $U_{T}$ is different from that used for $\left|U_{T}\right|$ in Figure 13. The top figure shows the examined area, indicated by a cyan rectangle in the engineering drawing.

Time-averaged distributions of $U_{T}\left(x,y\right)$ were obtained from the time-resolved TSP data via the TR approach. First, a set of $U_{T}\left(x,y\right)$ distributions was determined from successive maps of $T_{w}^{\prime }\left(x,y\right)$, i.e., with Δ*t* = 1/*f*_acq_ = 1 ms (see Section 2.2.2). The mean of the $U_{T}\left(x,y\right)$ maps was then calculated, and is shown in Figure 14. Note that, with the TR approach, the sign of the propagation celerity of temperature perturbations can also be determined, clearly showing a reverse flow region at approximately 16% < *x*/*c* < 22%. However, the $U_{T}\left(x,y\right)$ distribution obtained via the TR approach also showed spanwise non-uniformities in the region at approximately *y*/*c* < 0.4. These non-uniformities were likely caused by flow-independent temperature fluctuations (see Section 5.2.1). Moreover, a region of decreasing $U_{T}\left(x,y\right)$ can be seen in Figure 14 at approximately *x*/*c* > 27%. The apparently decreasing propagation celerity of temperature perturbations was probably due to the decreasing TSP signal-to-noise ratio in this region, which did not enable the appropriate tracking of the thermal traces. Thus, $U_{T}$ was observed to unphysically vanish at approximately *y*/*c* > 0.4, while it even became negative at approximately *y*/*c* < 0.4, where the combination of the two negative effects led to an unphysical reverse flow region.

In spite of these issues, flow separation and reattachment in the region at approximately 16% < *x*/*c* < 22% could be clearly detected over the whole considered span width by identifying the locations corresponding to $U_{T}$ = 0. The spanwise-averaged separation and reattachment locations were found at *x*_S_/*c* = 17.7% and *x*_R_/*c* = 20.6%, with a variation over the span of Δ(*x*_S_/*c*) = ±0.9% and Δ(*x*_R_/*c*) = ±0.6%. These findings were in line with the results obtained with the other approaches. A quantitative comparison with reference data of the critical locations determined via the three different approaches is presented in the next section.

### 5.3. Comparison of the Detected Critical Locations with Reference Data

For a quantitative validation of the results shown in Section 5.1 and Section 5.2, reference skin-friction data are available from an earlier experimental study [48], whereas numerical simulations were conducted in the present work to obtain further reference information. Both the experimental and numerical studies were carried out at the same test conditions as those considered in this work.

Skin-friction fields were estimated in [48] using the Global Luminescent Oil-Film Skin-Friction field Estimation method (GLOFSFE), which provided quantitative results in the attached laminar flow region, but only qualitative results in the other flow regions. Nevertheless, an estimation of the separation and reattachment locations can be obtained from the GLOF data presented in [48]. Although the critical lines and the areas in their vicinity were detected as regions of ill-posed nodes, the location where the laminar skin-friction profile approached zero (*x*_S_/*c* ~ 16.5%) can be reasonably identified as the separation location. Moreover, it was discussed in [48] that efficient oil-film removal occurred at the reattachment location, leading to a spanwise-oriented stripe with a very low oil-film thickness. The location of this stripe (i.e., the gray-masked stripe in the skin-friction distribution of [48]) can be reasonably assumed as the reattachment location (*x*_R_/*c* ~ 21.7%).

Laminar skin-friction profiles and separation locations were obtained via compressible boundary-layer calculations carried out using the laminar boundary-layer solver COCO [102]. In a manner similar to that described in [48], the surface pressure distributions from Figure 9 served as inputs for the boundary-layer computations, together with the freestream Mach number, the chord Reynolds number, and the freestream static temperature. For these simulations, the model surface was reasonably assumed to be an adiabatic wall, since the ratio between the heated- and adiabatic-wall temperatures *T_w_*/*T_aw_* was very close to 1. The computations were performed for both pressure distributions measured with the CNT and CFRP-inserts, to account for the aforementioned small differences in the region at approximately 15% ≤ *x*/*c* ≤ 25%. In fact, these led to different separation locations, which were predicted to occur at *x*_S_/*c* = 16.8% and 19.8% for the cases with the CNT and CFRP-inserts, respectively.

A comparison of the locations of flow separation and reattachment, identified in the present work via the TSP-based approaches, with the aforementioned reference data is presented in Table 1. The reported values of *x*_S_/*c* and *x*_R_/*c* were obtained from the TSP data analysis as spanwise averages in the region 0.43 ≤ *y*/*c* ≤ 0.53. This spanwise region was selected because the results presented in the previous sections showed that it was the least affected by the flow-independent issues. The reported uncertainties Δ(*x*_S_/*c*) and Δ(*x*_R_/*c*) are conservative estimations based on the variation of the critical locations in the same spanwise region. With respect to the very small boundary-layer thickness (*δ*_99_ ~ 350 µm upstream of the separation location, as predicted by the COCO computations) and to the small streamwise extent of the laminar separation bubble (a few percent of the chord), these uncertainties may appear relatively large; it should, however, be noted that the corresponding uncertainty in the LSB size is small (Δ(*x*_R_ – *x*_S_)/*c* < 1%), since the separation and reattachment lines were observed to be, in general, almost parallel. The separation and reattachment locations from the GLOFSFE data were estimated as spanwise averages over the region 0.375 ≤ *y*/*c* ≤ 0.625, with the related uncertainties estimated according to the information provided in [48]. The uncertainty in *x*_S_/*c* from the GLOFSFE data approximately corresponds to the streamwise region where the wall shear stress was estimated to be below $\tau_{w,x}$ ~ 2 Pa around the location of vanishing skin friction. The uncertainty in the estimated reattachment location approximately corresponds to the streamwise extent of the spanwise-oriented stripe with a very low oil-film thickness in the GLOFSFE data.

With the consideration of the measurement uncertainties and of the SWBLI-reproducibility aspects, all critical locations were in reasonable agreement, with a maximal deviation of Δ(*x*/*c*) = 3.3% (which is the difference in the separation locations from the GLOFSFE data and the COCO computations for the CFRP-insert). The separation and reattachment locations obtained on the CNT-insert via the three different TSP-based approaches were in mutual agreement, with a maximal deviation of Δ(*x*/*c*) = 2.2%. With regard to the separation locations, the smallest difference between the measurements and the numerical predictions for the CNT-insert was found with the TR approach (Δ(*x_s_*/*c*) = 0.6%), whereas the agreement for the CFRP-insert (OF approach vs. COCO) was excellent (Δ(*x_s_*/*c*) = 0.2%).

From the comparison presented in Table 1, all three TSP-based approaches appear to be suitable for the identification of separation and reattachment locations in transonic, high Reynolds number flows. Although the detection of the critical lines via the OF approach is still possible even in the presence of flow-independent temperature gradients (see Figure 11), the imposed heat flux should be as uniform as possible, in order to increase the accuracy of the results (such as in the case of the CFRP heating). On the other hand, the approaches based on the time-resolved TSP data are only marginally exposed to the influence of a non-uniform heat flux, but may be affected by its fluctuations.

### 5.4. Exploration of the Feasibility to Determine the Quantitative Skin-Friction Distribution via the OF and TR Approaches

The TSP-based approaches allow for the extraction of skin-friction fields over the examined surface. As discussed in the previous sections, in the present work they were however affected by flow-independent changes (spatial and/or temporal) in the temperature field. For this reason, the possibility to obtain quantitative skin-friction distributions was explored only for the spanwise region 0.43 ≤ *y*/*c* ≤ 0.53, in which the data were less affected by the aforementioned issues. Streamwise distributions of $\tau_{w,x}$ and $U_{T}$ were determined via the OF and TR approaches, respectively, and then averaged over this spanwise region. The results obtained via the TH approach were not considered in this exploration because of the observed saturation of $\left|U_{T}\right|$ (see Section 5.2.1).

In the case of the TR approach, the wall shear stress was obtained from the propagation celerity of temperature perturbations following a procedure based on the physical relationship between $U_{T}$ and $u_{\tau ,x}$ discussed in [25,57,75] (see Section 2.2). The distribution of an uncalibrated wall shear stress ${\hat{\tau }}_{w,x}$ was determined as:

(6) $${\hat{\tau }}_{w,x}=\rho_{\infty }U_{T}{}^{2}$$

where $\rho_{\infty }$ is the density of the freestream, determined from the freestream static pressure and temperature (see Section 3.1) according to the ideal gas law. The ${\hat{\tau }}_{w,x}$-distribution was then scaled by using an appropriate reference value of $\tau_{w,x}$, which was selected at *x*/*c* = 13% from the numerical data. The proportionality constant between $U_{T}$ and $u_{\tau ,x}$ would correspond, in this case, to 8.7. In contrast to the value used in incompressible flows [25,57,75], this value of the proportionality constant does not rely on the findings of earlier publications, but was merely determined via the aforementioned calibration procedure. Therefore, its physical reason must be investigated in future studies.

**Figure 15:**
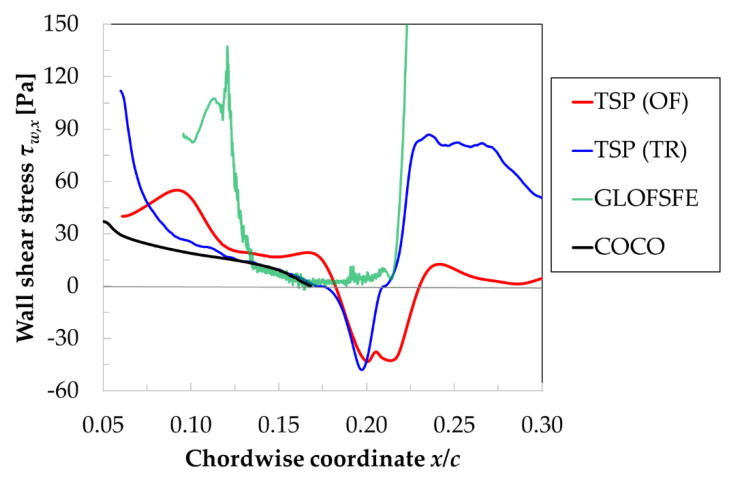
Distribution of the streamwise component of the wall shear stress estimated via the OF and TR approaches on the VA-2 airfoil model with the CNT-insert, investigated at M = 0.72, Re = 9.7 × 10^6^ and AoA = 1.5°. The numerical profile obtained using COCO and the experimental profile from [48] (GLOFSFE) are also shown.

The $\tau_{w,x}$–profiles obtained via the OF and TR approaches are shown in Figure 15, where they are also compared with the corresponding numerical and experimental reference distributions. As can be seen in this figure, the (calibrated) $\tau_{w,x}$–profile obtained via the TR approach was in excellent agreement with that computed via COCO in the laminar region 12% ≤ *x*/*c* ≤ 16.8%, i.e., up to the separation location. In the region at approximately 13.5% ≤ *x*/*c* ≤ 16.5%, these distributions were also in agreement with the $\tau_{w,x}$–profile obtained via GLOFSFE. As discussed in [48], this was also the only region where the GLOFSFE estimation could be regarded as quantitative: in the areas upstream of *x*/*c* ~ 13.5% and downstream of the location of transition onset, the skin friction was overestimated, whereas the recirculation region could not be captured via GLOFSFE. In contrast, the TR approach seemed to capture also the peak in (negative) skin friction within the laminar separation bubble, as well as the skin-friction increase immediately downstream of flow reattachment. The $\tau_{w,x}$–level in the turbulent region downstream of the reattachment location also appears reasonable, but no reference data are available for comparison in this region. In any case, the skin-friction decrease at approximately *x*/*c* > 27% was unphysical (see the related discussion in Section 5.2.2). The large values of $\tau_{w,x}$ in the region at *x*/*c* < 12% were also unphysical; they may be related to the different locations of the leading edges of the momentum and thermal boundary layers, which may have led to an apparently larger skin friction derived from the thermal data. This aspect will be the focus of future investigations.

The reason for the large skin-friction values obtained via the OF approach in the region at *x*/*c* < 12% may be the same, although in the region at *x*/*c* < 9% the decrease of $\tau_{w,x}$ in the upstream direction (i.e., towards the leading edge) was likely due to the decrease in the heating efficiency of the CNT layer (in the area close to the metal–TSP interface), which directly affected the skin-friction distribution in the OF approach. In the region at 12% ≤ *x*/*c* ≤ 15%, the wall shear stress obtained via the OF approach was close to that obtained via the TR approach and the COCO computations, but a local peak in $\tau_{w,x}$ was then found upstream of the separation location. The reason for this local peak has not been clarified yet, but it shows a further, even qualitative difference between the $\tau_{w,x}$–profile obtained via the OF approach and the distribution computed using COCO.

Concluding this section, it should be emphasized that both TSP-based approaches (OF and TR) were capable of capturing the reverse flow condition (negative wall shear stress) within the laminar separation bubble. This was not possible via COCO and GLOFSFE.

## 6. Conclusions and Recommendations for Future Work

This work focused on the further development and application of TSP-based methodologies for the global identification of flow separation and reattachment in compressible, high Reynolds number flows. The methodologies for the skin-friction extraction from TSP data, which had been developed for incompressible flows, were adapted in this work and applied to study a laminar separation bubble resulting from a shock-wave/boundary-layer interaction. The experiments were conducted in the Transonic Wind Tunnel Göttingen on a VA-2 supercritical airfoil model with a 1 m chord by a 1 m span. The investigations focused on the model upper surface, which was equipped with two exchangeable TSP inserts, specifically designed for transonic, high Reynolds number tests. In particular, two different types of electrical heating were integrated to the TSP layer composition: a layer of carbon nanotubes in the CNT-insert, and a current-carrying carbon fiber layer in the CFRP-insert. The TSP data were acquired with two different optical setups. In the case of the CNT-insert, the TSP images were recorded at a frequency of 1 kHz using a high-speed camera, which could capture only a portion of the TSP field; full-field TSP data were acquired with high spatial resolution on the CFRP-insert using two cameras, which allowed for a temporal resolution of only 3.3 Hz. Besides TSP, the model was also instrumented with pressure taps for the measurement of the surface pressure distribution, and with thermocouples to provide reference information on the surface temperature. The examined test conditions were a freestream Mach number of 0.72, a chord Reynolds number of 9.7 × 10^6^ and an angle of attack of 1.5°.

For the first time in a compressible flow, a laminar separation bubble (resulting from the SWBLI on the model upper surface) was detected via the TSP-based approaches. Three different approaches were considered. The OF approach, which is grounded on the recasting of the energy equation at the wall in a form similar to that of the optical flow equation, was adapted to compressible flows by using the adiabatic-wall temperature distribution as the appropriate reference temperature map for the method. By means of the OF approach, the topological maps of the skin-friction lines were obtained. This enabled the identification of flow separation and reattachment at the locations of, respectively, convergence and divergence of the skin-friction lines. The other two approaches are based on the relationship between the friction velocity and the propagation celerity of temperature perturbations $U_{T}$, which was determined from time-resolved TSP data. In the TH approach, the distribution of the modulus of $U_{T}$ was obtained by minimizing the dissimilarity between the measured evolution of the wall temperature perturbations (i.e., that of traceable passive scalars) and the behavior conforming to the Taylor hypothesis. The TR approach relies on the direct tracking of the wall temperature perturbations via an efficient optical flow algorithm, which allowed for the determination of the propagation celerity of temperature perturbations, including its sign. The minima of $\left|U_{T}\right|$ and the zero-crossing of $U_{T}$, obtained via the TH and TR approaches, respectively, also enabled the global identification of the critical lines.

The locations of flow separation and reattachment identified via the three TSP-based approaches were in mutual agreement, with maximal differences of the order of Δ(*x*/*c*) = 2%. The identification of the critical lines via the OF approach was found to be robust, since it allowed for the detection of flow separation and reattachment on the CNT-insert in spite of the influence of flow-independent gradients of the surface temperature, which were induced by a non-uniform surface heating and led to an unphysical distortion of the skin-friction lines. On the CFRP-insert, the imposed heat flux was more homogeneous, and the determined skin-friction lines were oriented in the streamwise direction for the whole laminar and reverse flow regions. However, in most of the turbulent region, the flow-independent temperature gradients did affect the topology of the skin-friction lines. This was due to the combination of the weakening of the streamwise temperature gradient and of the decrease in the TSP signal-to-noise ratio. The TH and TR approaches were only marginally exposed to the effect of a non-uniform imposed heat flux, but seemed to be affected by the influence of flow-independent temperature fluctuations. Moreover, the values of $\left|U_{T}\right|$ determined via the TH approach in the regions far from the laminar separation bubble appeared to saturate, likely because of the relatively low TSP data acquisition frequency, which was probably not fully adequate for the application of this approach in the considered high-speed, attached boundary layer.

In any case, the critical lines identified via the TSP-based approaches were shown to be in reasonable agreement with reference experimental and numerical data, which were obtained, respectively, via a global luminescent oil film in a previous experiment and via laminar boundary-layer computations in the present work. With the consideration of the experimental uncertainties, all reattachment locations were in agreement with the estimations from the GLOF data. Excellent agreement with the numerical separation locations was observed for the TR approach on the CNT-insert, and for the OF approach on the CFRP-insert.

The feasibility to obtain quantitative skin-friction distributions from the TSP data at the examined transonic flow conditions was also explored. The comparison of the spanwise-averaged $U_{T}$-distribution, determined via the TR approach, with the reference distributions of the streamwise component of the wall shear stress $\tau_{w,x}$ showed promising results. In particular, the experimental $\tau_{w,x}$–profile, obtained after calibration with a reference skin-friction value, was in agreement with the computational results for a portion of the laminar region, and also presented reasonable skin-friction levels upstream and downstream of the reattachment location. On the other hand, the $\tau_{w,x}$–distribution obtained via the OF approach was even qualitatively different from the reference profiles.

In summary, this work presented a first demonstration of TSP as a global sensor for the identification of the critical lines in compressible, high Reynolds number flows. Further studies are, however, needed for the consolidation of the TSP-based approaches at such challenging flow conditions, especially when aiming to the quantitative determination of the wall shear stress. Based on the observations reported in the present work, the following improvements should be considered as the next important steps:

- The imposed heat flux should be as homogeneous and stable as possible. As could be seen in this work, a current-carrying carbon fiber layer appears to be the most promising heating system to impose a uniform heat flux. As compared to the present results, a further improvement in the heat flux uniformity and stability can be achieved by applying a screening layer between the TSP and the current-carrying carbon fiber layers. This adaptation of the layer composition should lead to a compensation of possible heating inhomogeneities.
- The above improvement is expected to lead also to an increase of the signal-to-noise ratio in the turbulent flow region. Nevertheless, for the investigated test conditions with relatively small flow-induced temperature gradients, the surface temperature differences should be further enhanced, for example, by applying the highest electrical power allowed for the safe operation of the current-carrying carbon fiber layer.
- For the study of the potential and of the limits of the approaches relying on time-resolved TSP data (in particular, of the TH approach), it is recommended to perform measurements using a TSP with a shorter response time (e.g., based on a ruthenium complex [103,104]) and to record the TSP data at a higher acquisition frequency (*f*_acq_ of the order of 10 kHz).
- Reference data should also be generated for the turbulent flow region. As discussed in Section 1, the examined flow conditions are challenging for skin-friction measurements, but oil-film interferometry appears to be the most appropriate technique to obtain quantitative skin-friction data in the DNW-TWG, even in the turbulent flow region. It is also suggested to perform numerical simulations that can account for boundary-layer transition and SWBLI, in order to carry out comparisons between the numerical and the experimental results. Note, here, that not only the boundary layer developing on the model surface, but probably also the wind-tunnel environment [89] should be considered.

After the implementation of these improvements, future studies should focus on the exploration of the capabilities and limits of the TSP-based approaches in compressible flow scenarios which are different from that examined in the present work, such as cases involving more extended laminar separation bubbles, turbulent flow separation and/or three-dimensional structures in separated flow regions.

## Figures and Tables

**Table 1:** Consistency of the locations of flow separation (*x*_S_/*c*) and reattachment (*x*_R_/*c*), identified in the present work via TSP-based approaches as spanwise averages in the region 0.43 ≤ *y*/*c* ≤ 0.53, and comparison with experimental (GLOFSFE) [48] and numerical (COCO) reference data. The variables marked with “Δ” indicate the estimated uncertainties of the critical locations.

Data	*x*_S_/*c* [%]	Δ(*x*_S_/*c*) [%]	*x*_R_/*c* [%]	Δ(*x*_R_/*c*) [%]
OF approach (CNT-insert)	18.2	±0.7	23.0	±1.0
TH approach (CNT-insert)	19.6	±1.0	22.5	±1.0
TR approach (CNT-insert)	17.4	±0.7	21.0	±0.5
COCO (CNT-insert)	16.8	-	-	-
GLOFSFE [48]	16.5	±1.5	21.7	±1.0
OF approach (CFRP-insert)	19.6	±0.5	22.1	±0.7
COCO (CFRP-insert)	19.8	-	-	-

## Data Availability

The data presented in this study are available on request from the corresponding author.
